# Longitudinal care continuity and avoidable hospitalization: the application of claims-based measures

**DOI:** 10.1186/s12913-023-09457-w

**Published:** 2023-05-27

**Authors:** Shou-Hsia Cheng, Chi-Chen Chen, Yueh-Yun Lin

**Affiliations:** 1 Institute of Health Policy and Management, College of Public Health, National Taiwan University, Room 618, 17, Hsu-Chow Road, Taipei, 100 Taiwan; 2 Population Health Research Center, National Taiwan University, Taipei, Taiwan; 3 Department of Public Health, College of Medicine, Fu-Jen Catholic University, Taipei, Taiwan

**Keywords:** Continuity of care, Longitudinal, Duration, Claims-based, Indicator

## Abstract

**Background:**

Longitudinal continuity between a patient and his/her primary care physician is an important aspect in measuring continuity of care (COC). The majority of previous studies employed questionnaire surveys to patients to measure the continual relationship between patients and their physicians. This study aimed to construct a provider duration continuity index (PDCI) by using longitudinal claims data and to examine its agreement with commonly used COC measures. Then, this study investigated the effects of the various types of COC measure on the likelihood of avoidable hospitalization while considering the level of comorbidity.

**Methods:**

This study constructed a 4-year panel (from 2014 to 2017) of the nationwide health insurance claims data in Taiwan. In total, 328,044 randomly selected patients with 3 or more physician visits per year were analyzed. Two PDCIs were constructed to measure the duration of interaction between a patient and his/her physicians over time. The agreement between the PDCIs and three commonly used COC indicators, the Usual Provider of Care index, the Continuity of Care Index, and the Sequential Continuity Index, were examined. Generalized estimating equations were conducted to examine the association between COC and avoidable hospitalization by the level of comorbidity.

**Results:**

The results showed that the correlations among the three commonly used COC indicators were high (γ = 0.787 ~ 0.958) and the correlation between the two longitudinal continuity measures was moderate (γ = 0.577 ~ 0.579), but the correlations between the commonly used COC indicators and the two PDCIs were low (γ = 0.001 ~ 0.257). All COC measures, both the PDCIs and the three commonly used COC indicators, showed independent protective effects on the likelihood of avoidable hospitalization in three comorbidity groups.

**Conclusion:**

The duration of interaction between patients and physicians is an independent domain in measuring COC and has a significant effect on health care outcomes.

**Supplementary Information:**

The online version contains supplementary material available at 10.1186/s12913-023-09457-w.

## Introduction

Continuity of care (COC) is an essential element in the health care delivery system. Previous studies have reported that higher levels of COC are associated with better health care outcomes and lower health care spending [1]. Therefore, many countries have implemented strategies to improve COC, such as patient-centered medical homes [2]. To investigate the impacts of these programs, good tools for measuring COC are critical. However, the concept and measurement of COC remain challenging.

Researchers have conceptualized multiple dimensions of COC, including longitudinal [3–5], informational [4–6], interpersonal/relational [4–6] and managerial [6] dimensions. Longitudinal continuity of care represents the ongoing relationship between patients and their physician regardless of the presence or absence of particular problems or illnesses [3]. This ongoing relationship between patients and their physicians may lead to favorable health care outcomes by enhancing the exchange of knowledge and mutual trust. Two streams of study have examined the relationship between longitudinal continuity of care and health care outcomes. Among those studies focusing on the duration of the ongoing patient-physician relationship, the majority found that a longer duration was associated with improved patient trust in the physician [7, 8] and increased patient satisfaction [9, 10]. A longer patient-physician relationship was also associated with decreased use of outpatient specialist services and hospitalization [11, 12], lower health care expenses [11] and reduced risk for mortality [13, 14]. On the other hand, the longitudinal relationship between patient and physician usually depends upon whether the patient has a regular source of care, i.e., a provider or facility, where she/he receives most of the health care. The health care provider or team at the facility assumes responsibility for coordinating necessary services for the patient. The results showed that having a usual source of care was associated with improved receipt of preventive services [15, 16] and improved quality of medical care experiences [17]. According to the results of the abovementioned studies, longitudinal continuity of care alone shows an independent favorable effect on health care outcomes.

With regard to the measures of longitudinal continuity of care, the majority of previous studies employed questionnaire surveys to the patients [7–10, 15–17]. Due to the high cost of data collection, previous studies tended to be conducted on small sample sizes and in restricted locations and were not able to track the changes in longitudinal continuity of care over time. Along with the increasing availability of health insurance claims data, the majority of claims-based quantitative COC indicators measure the concentration, dispersion or sequence of a patient’s physician visits [18]; they imply that repeated or less-dispersed visits to certain physicians may represent better longitudinal continuity of care between patients and their physicians [5]. However, longitudinal continuity of care might not be equivalent to those quantitative COC indicators based on contact patterns with physicians.

Using a nationally representative sample, this study aimed to construct new longitudinal COC indicators and to examine the correlation between the longitudinal continuity of care indicators and commonly used quantitative continuity indicators. Furthermore, the effect of longitudinal continuity of care on avoidable hospitalization was evaluated while considering multiple comorbidities because such patients tend to have more visits to either the same or multiple physicians, thereby affecting continuity of care.

## Materials and methods

### Data source and study design

This study employed the NHI database from the Health and Welfare Data Science Center, Ministry of Health and Welfare in Taiwan. We randomly selected 1 million subjects from the entire NHI enrollee population at the end of 2007 and retrieved their health utilization records from the claims data in the following years. For the sample subjects, we were able to collect all of the health care utilization information between January 1, 2007, and December 31, 2018.

### Study population

**Fig. 1:**
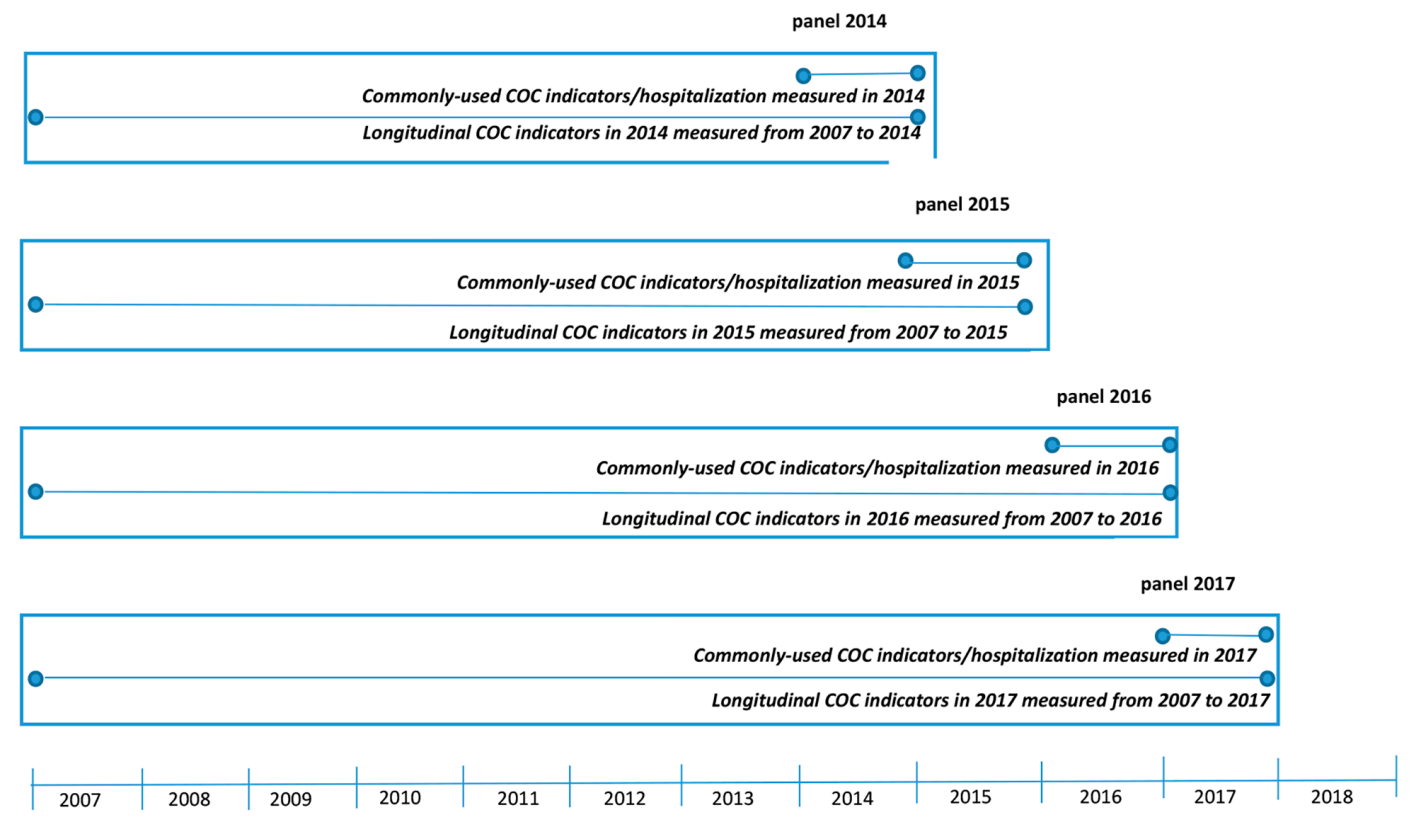
Measurement of the time periods for continuity of care and hospitalization for a typical patient

We included individuals if they (1) were 20 years of age or older in 2007; (2) were alive throughout the study period (from 2007 to 2018) to ensure comprehensive follow-up observations; and (3) had three physician visits or more each year during the study period (2007–2018) to allow meaningful COC scores to be obtained [19]. The present study was conducted using a panel study design with a 4-year panel of NHI claim records between 2014 and 2017 (Fig. 1). The feature of panel study design is the use of repeated measurement from the same subject over time [20]. The panel study design meets two goals; first, the panel study design can determine the change over time of the outcome measurements and the factors that influence the changes [20, 21], and second, the panel study design allows us to capture the unobserved time-invariant characteristics for one patient to another [20, 22]. We used the data from 2014 as baseline information and incorporated the data from the subsequent 4 years from 2014 to 2017. In other words, our analysis included four years of repeated measures of the individuals’ COC as well as their hospital admissions for avoidable conditions. In total, 328,044 subjects and 1,312,176 subject-years were included in this study. The unit of analysis was subject-years. In the present study, the Charlson Comorbidity Index (CCI), a score containing 17 categories of comorbid conditions defined by ICD-9-CM and ICD-10-CM codes [23], was employed. Patients were categorized into 3 mutually exclusive groups for each year according to the CCI (CCI = 0, CCI = 1 and CCI ≥ 2), and subject-years were then calculated for each group accounting for the dynamic changes in the CCI over the study period.

### Variable measures

#### Dependent variables

The main outcome variable was whether a subject had an avoidable hospitalization in each of the years from 2014 to 2017, which was coded as a dichotomous variable. Hospitalization for avoidable conditions or for ambulatory care-sensitive conditions has been used as a measure of the performance of primary care [24]. In this study, we employed the definition of avoidable hospitalization (Prevention Quality Indicator, PQI) published by the Agency for Healthcare Research and Quality [25]. The diagnoses for avoidable hospitalizations in our study included bacterial pneumonia, dehydration, pediatric gastroenteritis, urinary tract infection, perforated appendix, angina without procedure, congestive heart failure without specified cardiac procedure, hypertension, asthma, chronic obstructive pulmonary disease, and diabetes.

#### Independent variables

We focused on COC in outpatient settings only and used multiyear NHI claims data to develop a longitudinal continuity index, the provider duration continuity index (PDCI). We constructed two PDCIs for each year during the study period to indicate the continual relationship between a patient and his/her most frequently seen doctor. Based on the availability of the dataset from 2007 to 2018, we first used the first year panel (year 2014) as the end point and traced back all of the enrollees to the year 2007 with a period of 8-years of observation for PDCI. Then we used the panel of 2015 as the end point and traced back all of the enrollees to the year 2007 with a period of 9-years of observation. Similarly, we constructed the other two panels with observation period for 10 and 11 years, respectively. By doing so, we can maximize the use of the data longitudinally and to examine the stability of the PDCI calculation in the 4 panels (Fig. 1). For example, we consider the first year of the panel (year 2014): (1) PDCI_1_: We identified the physician who a patient visited most frequently in 2014 and calculated the duration of their interactions separately from 2007 to 2014. If more than one doctor had the same highest number of outpatient visits in 2014, the doctor who had a longer relationship with the patient was considered the most frequently seen physician. (2) PDCI_2_: We identified all of the physicians that a patient visited in 2014 and traced back the durations of the interactions between each physician and the patient from 2007 to 2014. The longest duration a patient visited with a specific physician was considered the PDCI_2_ for the patient. The computation of the PDCIs in the other years of the panel was the same as that for the first year of the panel. The value of the PDCIs ranged from 1 to 8 in the first year of the panel (as it spanned 2007 to 2014), 1 to 9 in the second year of the panel (2007 to 2015), 1 to 10 in the third year of the panel (2007 to 2016) and 1 to 11 in the last year of the panel (2007 to 2017). The index represents the duration in years of the relationship between a patient and his/her most frequently seen doctor during the observation period.

This study selected three commonly used claims-based COC measures, including “density type: usual provider of care (UPC) index”, “dispersion type: continuity of care index (COCI)”, and “sequence type: Sequential Continuity (SECON) index” [18] (Supplementary Table 1). The scores of these three commonly used COC measures were calculated at the physician level from 2014 to 2017 with a range between 0 and 1, with higher COC scores corresponding to better COC. The scores of all the COC measures were categorized into three equal tertiles according to the distribution across the entire study population.

#### Covariates

Several confounding characteristics were controlled for in the regression models, including time-varying variables and time-invariant variables. The time-varying variables in the models were age, the number of outpatient visits, the likelihood of hospitalization in the previous year, low-income status and rural-urban designation. Low-income status for a subject was identified by the status recorded in the NHI database. The rural-urban designation was based on the population density of the township; townships that fell in the lowest 20% according to population density were defined as rural areas in Taiwan. Patient sex was the time-invariant variable. Finally, the study included 3-year dummy variables, with 2014 as the reference group, to control for characteristics that may change over time.

### Statistical analyses

The Pearson correlation coefficient was employed to investigate the relationship between the three most frequently used claims-based COC indicators and the claims-based longitudinal measures (PDCIs). In addition, some unobserved patient characteristics, such as health care seeking behavior, might simultaneously affect COC and avoidable hospitalizations. Thus, we employed generalized estimating equations (GEEs) by using a longitudinal technique that could take the intraclass correlation between repeated observations for the same patients into account [20]. We used a binary distribution with a logit link for the dependent variables.

## Results

### Descriptive analyses

The characteristics of the study patients in 2014 are given in Table 1, categorized into CCI groups of CCI = 0, CCI = 1 and CCI ≥ 2. There were fewer male than female patients in each of the three CCI score groups. The mean numbers of physician visits among the CCI = 0, CCI = 1 and CCI ≥ 2 groups were 19.10, 29.63 and 37.79, respectively. The percentages of patients hospitalized in the previous year were 8.19%, 13.33% and 24.39% among the CCI = 0, CCI = 1 and CCI ≥ 2 groups, respectively.

**Table 1:** Baseline characteristics of the study sample in 2014

Characteristics	CCI = 0 (N = 208,505)			CCI = 1 (N = 91,170)			CCI ≥ 2 (N = 28,369)	
	N	%		N	%		N	%
Sex (N, %)								
Male	75,008	35.97		41,351	45.36		12,787	45.07
Female	133,171	63.87		49,733	54.55		15,563	54.86
Missing	326	0.16		86	0.09		19	0.07
Age (mean, SD)	51.43	14.55		62.11	13.17		64.82	12.43
Rural area (N, %)	43,481	20.85		19,545	21.44		4,884	17.22
Low income family (%)	1,982	0.95		1,108	1.22		351	1.24
Physician visits (mean, SD)	19.10	13.85		29.63	18.37		37.79	22.48
Physician visits (N, %)								
Low	95,246	45.68		16,034	17.59		2,770	9.76
Intermediate	65,246	31.29		30,237	33.17		6,697	23.61
High	48,013	23.03		44,899	49.25		18,902	66.63
Hospitalization in the previous year (N, %)	17,067	8.19		12,152	13.33		6,920	24.39

CCI, charlson comorbidity index

The variables of interest are given in Table 2. With regard to the longitudinal continuity of care indicators, the mean value of PDCI_1_ in year 2014 (from 2007 to 2014), year 2015 (from 2007 to 2015), year 2016 (from 2007 to 2016) and year 2017 (from 2007 to 2017) was 4.74 years, 5.13 years, 5.50 years and 5.81 years, respectively in the CCI = 0 group. Similar trends were observed among the other CCI groups. In addition, the values of the three commonly used COC indicators remained stable from 2014 to 2017 among the three CCI groups.

**Table 2:** Main interest variable according to the year

	Year 2014		Year 2015		Year 2016		Year 2017	
**CCI = 0**								
COC (mean, SD)								
Longitudinal continuity indicators								
PDCI_1_	4.74	2.59	5.13	2.93	5.50	3.27	5.81	3.60
PDCI_2_	6.23	1.95	6.83	2.26	7.43	2.55	7.96	2.88
Claims-based COC indicators								
UPC index	0.47	0.21	0.47	0.21	0.47	0.21	0.47	0.21
COCI	0.29	0.23	0.29	0.23	0.29	0.23	0.29	0.23
SECON index	0.38	0.24	0.38	0.24	0.38	0.24	0.39	0.24
Hospitalization for avoidable conditions (N, %)	992	0.48	1,068	0.53	1,286	0.63	1,246	0.63
**CCI = 1**								
COC (mean, SD)								
Longitudinal continuity indicators								
PDCI_1_	5.09	2.58	5.52	2.93	5.90	3.28	6.22	3.62
PDCI_2_	6.75	1.71	7.43	2.00	8.09	2.31	8.69	2.64
Claims-based COC indicators								
UPC index	0.48	0.21	0.48	0.21	0.47	0.21	0.47	0.21
COCI	0.32	0.22	0.32	0.22	0.32	0.22	0.32	0.22
SECON index	0.44	0.22	0.43	0.22	0.43	0.22	0.43	0.22
Hospitalization for avoidable conditions (N, %)	2,130	2.34	2,282	2.44	2,605	2.83	2,641	2.81
**CCI ≥ 2**								
COC (mean, SD)								
Longitudinal continuity indicators								
PDCI_1_	4.89	2.57	5.26	2.90	5.57	3.24	5.84	3.56
PDCI_2_	6.86	1.65	7.56	1.93	8.22	2.24	8.83	2.56
Claims-based COC indicators								
UPC index	0.42	0.18	0.42	0.18	0.41	0.18	0.41	0.18
COCI	0.27	0.18	0.27	0.18	0.26	0.17	0.26	0.17
SECON index	0.39	0.19	0.39	0.19	0.39	0.18	0.39	0.18
Hospitalization for avoidable conditions (N, %)	1,241	4.37	1,486	4.74	1,646	5.12	1,937	5.36

CCI, charlson comorbidity index; COC, continuity of care; UPC index, usual provider of care index; COCI, continuity of care index; SECON index, sequential continuity index; PDCI, provider duration continuity index

### Correlation between PDCIs and commonly used COC measures

Table 3 presents the Pearson’s correlation coefficients among the COC measures. We found that the three commonly used COC indicators were highly correlated within the individual study periods. For the longitudinal COC indicators, the correlation between the two PDCIs was moderate (γ = 0.577). Conversely, the longitudinal COC indicators, both PDCI_1_ and PDCI_2_, were weakly correlated with the three commonly used COC indicators (γ = 0.022–0.257). Similar results were observed in the other panel years.

**Table 3:** Correlation between the longitudinal provider duration continuity measures and three commonly used continuity of care indexes according to year

	Longitudinal continuity of care indicators			Commonly used continuity of care indicators		
	PDCI_1_	PDCI_2_		UPC	COCI	SECON index
**Year 2014**						
Longitudinal continuity indicators						
PDCI_1_	1.000	0.577		-	-	-
PDCI_2_	-	1.000		-	-	-
Claims-based COC indicators						
UPC index	0.232	0.022		1.000	0.958	0.794
COCI	0.257	0.068		-	1.000	0.846
SECON index	0.166	0.050		-	-	1.000
**Year 2015**						
Longitudinal continuity indicators						
PDCI_1_	1.000	0.579		-	-	-
PDCI_2_	-	1.000		-	-	-
Claims-based COC indicators						
UPC index	0.230	0.017		1.000	0.958	0.792
COCI	0.253	0.062		-	1.000	0.845
SECON index	0.164	0.044		-	-	1.000
**Year 2016**						
Longitudinal continuity indicators						
PDCI_1_	1.000	0.579		-	-	-
PDCI_2_	-	1.000		-	-	-
Claims-based COC indicators						
UPC index	0.228	0.011		1.000	0.958	0.788
COCI	0.252	0.057		-	1.000	0.842
SECON index	0.165	0.042		-	-	1.000
**Year 2017**						
Longitudinal continuity indicators						
PDCI_1_	1.000	0.578		-	-	-
PDCI_2_	-	1.000		-	-	-
Claims-based COC indicators						
UPC index	0.222	0.001		1.000	0.957	0.787
COCI	0.244	0.045		-	1.000	0.841
SECON index	0.160	0.033		-	-	1.000

UPC index, usual provider of care index; COCI, continuity of care index; SECON index, sequential continuity index; PDCI, provider duration continuity index

### Effects of PDCIs and commonly used COC measures on avoidable hospitalization

Two sets of results are obtained, one for the effects of PDCI_1_ and each of three commonly used COC measures (Model 1a: PDCI_1_ and UPC index; Model 1b: PDCI_1_ and COCI; Model 1c: PDCI_1_ and SECON index); another for the PDCI_2_ and the each of three commonly used COC measures (Model 2a- 2c). Significant dose-response effects were observed for both the longitudinal COC measures and the commonly used COC measures on the likelihood of avoidable hospitalization (Table 4).

**Table 4:** GEE estimations of the effects of the longitudinal COC indicators and claims-based COC measures on the likelihood of hospitalization for avoidable conditions by CCI group

	CCI = 0				CCI = 1				CCI ≥ 2		
	OR	95% CI			OR	95% CI			OR	95% CI	
**Model 1a: PDCI 1 and UPC index**											
PDCI _1_(reference group: low)											
Intermediate	0.85	0.79	0.92		0.77	0.73	0.81		0.80	0.75	0.85
High	0.80	0.74	0.86		0.67	0.63	0.70		0.72	0.67	0.76
UPC index (reference group: low)											
Intermediate	0.78	0.72	0.83		0.86	0.82	0.90		0.74	0.7	0.79
High	0.59	0.55	0.64		0.67	0.63	0.72		0.64	0.59	0.7
**Model 1b:PDCI 1 and COCI**											
PDCI _1_(reference group: low)											
Intermediate	0.87	0.81	0.93		0.77	0.74	0.82		0.8	0.75	0.85
High	0.83	0.77	0.89		0.68	0.64	0.71		0.73	0.68	0.78
COCI (reference group: low)											
Intermediate	0.74	0.69	0.79		0.81	0.77	0.85		0.68	0.64	0.72
High	0.51	0.47	0.56		0.62	0.58	0.66		0.55	0.51	0.6
**Model 1c:PDCI 1 and SECON index**											
PDCI _1_(reference group: low)											
Intermediate	0.84	0.78	0.90		0.76	0.72	0.80		0.79	0.74	0.84
High	0.77	0.71	0.83		0.66	0.62	0.69		0.71	0.66	0.76
SECON index (reference group: low)											
Intermediate	0.84	0.79	0.90		0.84	0.80	0.88		0.81	0.76	0.86
High	0.61	0.56	0.66		0.62	0.58	0.65		0.59	0.55	0.64
**Model 2a:PDCI 2 and UPC index**											
PDCI _2_(reference group: low)											
Intermediate	0.80	0.74	0.86		0.73	0.69	0.78		0.74	0.69	0.8
High	0.73	0.67	0.79		0.65	0.62	0.69		0.67	0.62	0.71
UPC index (reference group: low)											
Intermediate	0.77	0.72	0.83		0.84	0.80	0.88		0.72	0.68	0.77
High	0.58	0.54	0.63		0.63	0.59	0.67		0.6	0.55	0.65
**Model 2b: PDCI 2 and COCI**											
PDCI _2_(reference group: low)											
Intermediate	0.79	0.73	0.86		0.73	0.69	0.77		0.73	0.68	0.78
High	0.74	0.68	0.80		0.65	0.62	0.69		0.66	0.62	0.71
COCI (reference group: low)											
Intermediate	0.73	0.69	0.79		0.79	0.75	0.83		0.66	0.63	0.70
High	0.50	0.46	0.54		0.57	0.54	0.61		0.52	0.48	0.56
**Model 2c: PDCI 2 and SECON index**											
PDCI _2_(reference group: low)											
Intermediate	0.79	0.73	0.85		0.73	0.69	0.77		0.74	0.68	0.80
High	0.70	0.65	0.76		0.64	0.60	0.67		0.66	0.62	0.71
SECON index (reference group: low)											
Intermediate	0.84	0.79	0.90		0.83	0.79	0.87		0.8	0.76	0.85
High	0.59	0.55	0.64		0.59	0.55	0.62		0.57	0.52	0.61

CCI, charlson comorbidity index; COC, continuity of care; UPC index, usual provider of care index; COCI, continuity of care index; SECON index, sequential continuity index; PDCI, provider duration continuity index

All models were controlled for sex, age, rural area, low income family, physician visits, hospitalization in the previous year

### Sensitivity analyses

In addition to these models, we conducted three sensitivity analyses to improve the robustness of this study. First, the analyses using outcome measures in the subsequent years also indicated significant dose-response effects of the commonly used COC measures and PDCI_2_ on the likelihood of avoidable hospitalization, but none were seen for PDCI_1_ (Supplementary Table 2; Supplementary Fig. 1). Second, we also investigated the effects of COC on avoidable hospitalization based on the definition provided by the **Institute of Medicine** as well as on hospitalization for any conditions, and the findings remained almost unchanged when compared to those presented above (Supplementary Tables 3 and Supplementary Table 4) [26]. Finally, we performed analyses using stratified age groups (20–39 years, 40–59 years and ≥ 60 years) and found similar results to those described previously, except for patients in the younger age group (20–39 years) (Supplementary Table 5).

## Discussion

### Statement of principal findings

In this study, we found that the correlation between the PDCIs and three commonly used quantitative COC measures based on contact patterns with physicians was low. However, the results revealed independent effects of the PDCIs and the commonly used quantitative COC indicators on the likelihood of avoidable hospitalizations across all CCI groups.

### Strengths and limitations

Previous researchers considered longitudinal duration between patients and their physician to be a dimension of COC [3–5]. However, only a few studies have constructed longitudinal COC indicators by using claims data, such as specific COC measures that involve the density of care based on year-to-year follow-up [27, 28]. To the best of our knowledge, the PDCI in this study is the first indicator developed to measure the provider duration continuity by using administrative claims data. In health care systems with universal health coverage or containing complete patient health information for multiple years, calculating the number of years of continuity between a patient and a continuously visited physician with claims data is a feasible way to measure the provider duration continuity in addition to the traditional but costly survey.

The limitations of this study should be mentioned. First, we developed the longitudinal continuity measures by using claims data with a limited scope. Therefore, we are uncertain whether the provider duration continuity indicators reflect conditions of the patient-physician relationships, such as interpersonal continuity [5, 6]. Second, we did not include unobserved characteristics (such as health literacy) or unavailable variables (such as socioeconomic status and health status). However, this concern might have been mitigated because we used a panel study design accounting for time-invariant characteristics. Third, this study selected patients who were healthy enough to survive between 2007 and 2018, which might lead to an overestimation of the association between continuity of care and the likelihood of avoidable hospitalization in this study. Finally, this study employed claims data for patients 20 years of age and older with at least three physician visits every year during the study period, which excluded younger and healthier patients in the analysis; the results might not be generalizable to all residents of Taiwan.

### Interpretation within the context of the wider literature

In this study, the values of PDCI_1_ ranged from 5.81 to 6.22 years, and those PDCI_2_ ranged from 7.96 to 8.83 years in 2017. Is the length of the patient-physician relationship comparable to that in other health systems? Weiss (1996) [11] and Donahue et al. (2005) [10] revealed that over half of the elderly or rural population reported having a relationship with their physicians for more than five years. Mainous (2001) found that a total of 69.8% of UK adult patients and 8% of US adult patients had their regular physician for more than six years [8]. This study revealed that the longitudinal relationship between the patients and their most frequently seen physicians in a health system without formal referral arrangement appears to be no worse than that of other health systems.

In a health system with a gatekeeper or family doctor arrangement, we believe that the most frequently visited physician should be the gatekeeper or the family physician; therefore, PDCI_1_ may be an appropriate index representing the longitudinal continuity between a patient and his/her physician. On the other hand, in health systems without a gatekeeper or family physician, such as Taiwan, patients have excessive physician visits (approximately 13–15 visits per person per year) and visit multiple physicians in various health care institutions according to their preference [29]. Thus, PDCI_2_ may be more appropriate; using the greatest number of years between a patient and the physician who he/she has continuously visited to represent longitudinal continuity may be reasonable.

Interestingly, we found that the correlations between PDCIs and the three commonly used quantitative COC indicators were quite low. Unsurprisingly, the three claims-based measures captured the point estimates of a patient’s contact patterns with his/her physician, and the PDCIs measured the duration of visit encounters from a longitudinal perspective. Although the three commonly used quantitative COC indicators represent various types of COC, that is, the density (UPC), dispersion (COCI), and sequence (SECON index) of physician visits [18], the results from our study showed that the three commonly used indicators were highly correlated, which is consistent with previous studies [30, 31]. We recommend that investigators using claims data for research select a PDCI and a commonly used quantitative COC indicator simultaneously in their studies, which may better represent the various dimensions of COC of a patient.

With regard to the effects of longitudinal continuity of care on avoidable hospitalizations, we also found that higher PDCI scores were associated with a lower likelihood of avoidable hospitalization. Patients with a longer relationship with their physicians were less likely to be hospitalized for any condition as well as for avoidable conditions. The results are consistent with those reported from previous studies using survey methods [11, 12]. On the other hand, among the three commonly used quantitative COC indicators, we found that higher COC scores were associated with a lower likelihood of hospitalization for avoidable conditions. These findings are consistent with recent systematic reviews [1]. Furthermore, all three commonly used quantitative COC measures showed similar results, which suggests that the results concerning the effect of COC on avoidable hospitalizations are consistent regardless of the indicators selected.

Patients with multiple chronic conditions usually have complex care needs and receive treatment from several specialists in various health care institutions, which might deteriorate the COC between the patients and their physicians. Previous studies indicated that the effect of better COC on the reduction in duplicated medications [32] and drug-drug interactions [33] is more significant for patients with higher levels of comorbidity in the general population. In the present study, a longer duration for the relationship between the patients and their physicians was significantly associated with fewer avoidable hospitalizations across all three CCI comorbidity groups.

### Implications for policy, practice and research

In this study, we suggest that the PDCI is an appropriate and less costly indicator for measuring longitudinal continuity for researchers in this field. Even in a health system without formal referral requirements or gatekeepers, improving the longitudinal duration between patients and their physicians is beneficial both for the patients and for the health system.

## Conclusion

In this study, the results showed that the PDCIs were weakly correlated with the abovementioned COC indicators and that, similar to the commonly used COC indicators, the PDCIs were independent significantly associated with avoidable hospitalization across all comorbidity groups.

## Electronic supplementary material

Below is the link to the electronic supplementary material.

Supplementary Table 1 Commonly used claims-based COC measures. Supplementary Table 2 GEE estimations of the effects of the COC measures on the likelihood of hospitalization for avoidable conditions in the subsequent year by CCI group. Supplementary Table 3 GEE estimations of the effects of the COC measures on the likelihood of hospitalization for avoidable conditions using IOM definitions by CCI groupSupplementary Table 4 GEE estimations of the effects of the COC measures on the likelihood of hospitalization for any condition by CCI group. Supplementary Table 5 GEE estimations of the effects of the COC measures on the likelihood of hospitalization for avoidable conditions by age group. Supplementary Figure 1. Measurement of the time period for care continuity and hospitalization for a typical patient in the subsequent-year model.

## Data Availability

The data that support the findings of this study are available from the Health and Welfare Data Science Center in Taiwan. But restrictions apply to the availability of these data, which were used under license for the current study and therefore are not publicly available. However, available from the corresponding author (SH Cheng) upon reasonable request and with the permissions of the Health and Welfare Data Science Center.
