# Supplementary material for: Longitudinal care continuity and avoidable hospitalization: the application of claims-based measures
Source: BMC Health Serv Res. 2023 May 27;23:554. doi: 10.1186/s12913-023-09457-w (PMC10224272; doi:10.1186/s12913-023-09457-w)
Supplement: Supplementary file 1 — Supplementary Table 1 Commonly used claims-based COC measures. Supplementary Table 2 GEE estimations of the effects of the COC measures on the likelihood of hospitalization for avoidable conditions in the subsequent year by CCI group. Supplementary Table 3 GEE estimations of the effects of the COC measures on the likelihood of hospitalization for avoidable conditions using IOM definitions by CCI groupSupplementary Table 4 GEE estimations of the effects of the COC measures on the likelihood of hospitalization for any condition by CCI group. Supplementary Table 5 GEE estimations of the effects of the COC measures on the likelihood of hospitalization for avoidable conditions by age group. Supplementary Figure 1. Measurement of the time period for care continuity and hospitalization for a typical patient in the subsequent-year model. [file 12913_2023_9457_MOESM1_ESM.docx]

Supplementary Table 1 Commonly used claims-based COC measures

| Measure | Definition | Equation |
| --- | --- | --- |
| Usual Provider Continuity index (UPC index) (Breslau & Reeb, 1975) | The number of physician visits to the most frequently seen physician divided by the total number of physician visits. The index ranges from zero to one, with a greater value corresponding to greater care continuity. | ${UPC \mathrm{index}}_{p}=\frac{n_{p,m}}{N_{p}}$  where p represents a given patient*, N_p_* represents the total number of physician visits for patient p, and *n_p, m_* is the number of visits to the most frequently seen physician. |
| Continuity of Care Index (COCI) (Bice & Boxerman, 1977) | Composing of the number of different physicians seen and the number of visits to each physician. The value of COCI ranges from zero to one, with a greater value representing greater care continuity. | $COCI_{p}=\frac{\sum_{j=1}^{M_{p}} n_{p,j}^{2}-N_{p}}{N_{p}(N_{p}-1)}$  where p represents a given patient*, N_p_* represents the total number of physician visits for patient p, *n_p,j_* is the number of visits to the same physician *j*, *j* represents a given physician, and *M_p_* is the total number of physicians for patient p. |
| Sequential Continuity Index (SECON index) (Steinwachs, 1979) | Measuring the sequences of physicians being visited. The value ranges from zero to one, with a greater value representing better care continuity. | ${SECON}_{p}=\frac{\sum_{i=1}^{N_{p}-1} S_{p,i}}{N_{p}-1}$  where p represents a given patient; *N_p_* represents the total number of physician visits for patient p, which generates N_p_-1 sequential pairs of visits; S_p,i_ represents whether the patients have seen the same physician for sequential visits; s_p,i_=1 means if i visit and i+1 visit are to the same physician; and s_p,i_=0 represents otherwise. |

Supplementary Table 2 GEE estimations of the effects of the COC measures on the likelihood of hospitalization for avoidable conditions in the subsequent year by CCI group

|  | **CCI=0** | | |  | **CCI=1** | | |  | **CCI≥2** | | |
| --- | --- | --- | --- | --- | --- | --- | --- | --- | --- | --- | --- |
|  | OR | 95% CI | |  | OR | 95% CI | |  | OR | 95% CI | |
| **Model 1a: PDCI 1 and UPC index** |  |  |  |  |  |  |  |  |  |  |  |
| PDCI _1_(reference group: low) |  |  |  |  |  |  |  |  |  |  |  |
| Intermediate | 0.95 | 0.9 | 1.02 |  | 0.99 | 0.94 | 1.04 |  | 0.96 | 0.9 | 1.02 |
| High | 0.88 | 0.83 | 0.93 |  | 0.83 | 0.79 | 0.87 |  | 0.85 | 0.79 | 0.91 |
| UPC index (reference group: low) |  |  |  |  |  |  |  |  |  |  |  |
| Intermediate | 1.01 | 0.95 | 1.08 |  | 0.95 | 0.91 | 1 |  | 0.85 | 0.8 | 0.9 |
| High | 1.01 | 0.94 | 1.07 |  | 0.95 | 0.9 | 1.01 |  | 0.83 | 0.76 | 0.9 |
| **Model 1b:PDCI 1 and COCI** |  |  |  |  |  |  |  |  |  |  |  |
| PDCI _1_(reference group: low) |  |  |  |  |  |  |  |  |  |  |  |
| Intermediate | 0.96 | 0.9 | 1.02 |  | 0.99 | 0.94 | 1.04 |  | 0.96 | 0.9 | 1.02 |
| High | 0.89 | 0.83 | 0.94 |  | 0.83 | 0.79 | 0.87 |  | 0.85 | 0.79 | 0.91 |
| COCI (reference group: low) |  |  |  |  |  |  |  |  |  |  |  |
| Intermediate | 0.99 | 0.93 | 1.05 |  | 0.96 | 0.91 | 1.01 |  | 0.88 | 0.82 | 0.93 |
| High | 0.96 | 0.9 | 1.03 |  | 0.96 | 0.9 | 1.01 |  | 0.84 | 0.78 | 0.91 |
| **Model 1c:PDCI 1 and SECON index** |  |  |  |  |  |  |  |  |  |  |  |
| PDCI _1_(reference group: low) |  |  |  |  |  |  |  |  |  |  |  |
| Intermediate | 0.95 | 0.9 | 1.02 |  | 0.99 | 0.94 | 1.04 |  | 0.96 | 0.9 | 1.02 |
| High | 0.88 | 0.83 | 0.94 |  | 0.83 | 0.79 | 0.87 |  | 0.85 | 0.79 | 0.9 |
| SECON index (reference group: low) |  |  |  |  |  |  |  |  |  |  |  |
| Intermediate | 0.92 | 0.86 | 0.98 |  | 0.85 | 0.81 | 0.9 |  | 0.83 | 0.78 | 0.88 |
| High | 0.91 | 0.85 | 0.97 |  | 0.83 | 0.78 | 0.87 |  | 0.75 | 0.7 | 0.81 |
| **Model 2a:PDCI 2 and UPC index** |  |  |  |  |  |  |  |  |  |  |  |
| PDCI _2_(reference group: low) |  |  |  |  |  |  |  |  |  |  |  |
| Intermediate | 0.83 | 0.77 | 0.88 |  | 0.8 | 0.75 | 0.84 |  | 0.83 | 0.77 | 0.9 |
| High | 0.81 | 0.76 | 0.87 |  | 0.74 | 0.7 | 0.78 |  | 0.79 | 0.73 | 0.84 |
| UPC index (reference group: low) |  |  |  |  |  |  |  |  |  |  |  |
| Intermediate | 1.01 | 0.95 | 1.08 |  | 0.94 | 0.89 | 0.98 |  | 0.84 | 0.79 | 0.89 |
| High | 0.99 | 0.93 | 1.05 |  | 0.92 | 0.87 | 0.97 |  | 0.8 | 0.74 | 0.87 |
| **Model 2b: PDCI 2 and COCI** |  |  |  |  |  |  |  |  |  |  |  |
| PDCI _2_(reference group: low) |  |  |  |  |  |  |  |  |  |  |  |
| Intermediate | 0.83 | 0.77 | 0.88 |  | 0.8 | 0.75 | 0.84 |  | 0.84 | 0.77 | 0.9 |
| High | 0.82 | 0.77 | 0.87 |  | 0.74 | 0.7 | 0.78 |  | 0.79 | 0.73 | 0.85 |
| COCI (reference group: low) |  |  |  |  |  |  |  |  |  |  |  |
| Intermediate | 0.98 | 0.92 | 1.05 |  | 0.94 | 0.9 | 0.99 |  | 0.86 | 0.81 | 0.92 |
| High | 0.95 | 0.89 | 1.01 |  | 0.92 | 0.87 | 0.98 |  | 0.81 | 0.75 | 0.88 |
| **Model 2c: PDCI 2 and SECON index** |  |  |  |  |  |  |  |  |  |  |  |
| PDCI _2_(reference group: low) |  |  |  |  |  |  |  |  |  |  |  |
| Intermediate | 0.82 | 0.77 | 0.88 |  | 0.79 | 0.74 | 0.83 |  | 0.83 | 0.77 | 0.9 |
| High | 0.81 | 0.76 | 0.87 |  | 0.73 | 0.69 | 0.77 |  | 0.78 | 0.73 | 0.84 |
| SECON index (reference group: low) |  |  |  |  |  |  |  |  |  |  |  |
| Intermediate | 0.92 | 0.86 | 0.98 |  | 0.85 | 0.8 | 0.89 |  | 0.83 | 0.78 | 0.88 |
| High | 0.89 | 0.84 | 0.95 |  | 0.8 | 0.76 | 0.84 |  | 0.73 | 0.68 | 0.79 |

CCI, Charlson comorbidity index; COC, continuity of care; UPC index, usual provider of care index; COCI, continuity of care index; SECON index, sequential Continuity Index; PDCI, provider duration continuity index

Supplementary Table 3 GEE estimations of the effects of the COC measures on the likelihood of hospitalization for avoidable conditions using IOM definitions by CCI group

|  | **CCI=0** | | |  | **CCI=1** | | |  | **CCI≥2** | | |
| --- | --- | --- | --- | --- | --- | --- | --- | --- | --- | --- | --- |
|  | OR | 95% CI | |  | OR | 95% CI | |  | OR | 95% CI | |
| **Model 1a: PDCI 1 and UPC index** |  |  |  |  |  |  |  |  |  |  |  |
| PDCI _1_(reference group: low) |  |  |  |  |  |  |  |  |  |  |  |
| Intermediate | 0.83 | 0.79 | 0.88 |  | 0.77 | 0.73 | 0.81 |  | 0.82 | 0.77 | 0.87 |
| High | 0.75 | 0.71 | 0.80 |  | 0.69 | 0.66 | 0.73 |  | 0.74 | 0.70 | 0.79 |
| UPC index (reference group: low) |  |  |  |  |  |  |  |  |  |  |  |
| Intermediate | 0.76 | 0.72 | 0.81 |  | 0.82 | 0.79 | 0.86 |  | 0.73 | 0.69 | 0.77 |
| High | 0.60 | 0.56 | 0.64 |  | 0.62 | 0.58 | 0.66 |  | 0.59 | 0.54 | 0.64 |
| **Model 1b:PDCI 1 and COCI** |  |  |  |  |  |  |  |  |  |  |  |
| PDCI _1_(reference group: low) |  |  |  |  |  |  |  |  |  |  |  |
| Intermediate | 0.84 | 0.80 | 0.89 |  | 0.78 | 0.74 | 0.81 |  | 0.83 | 0.78 | 0.88 |
| High | 0.78 | 0.73 | 0.82 |  | 0.70 | 0.67 | 0.74 |  | 0.75 | 0.71 | 0.80 |
| COCI (reference group: low) |  |  |  |  |  |  |  |  |  |  |  |
| Intermediate | 0.74 | 0.70 | 0.79 |  | 0.78 | 0.75 | 0.82 |  | 0.68 | 0.64 | 0.72 |
| High | 0.54 | 0.50 | 0.57 |  | 0.56 | 0.53 | 0.60 |  | 0.51 | 0.47 | 0.56 |
| **Model 1c:PDCI 1 and SECON index** |  |  |  |  |  |  |  |  |  |  |  |
| PDCI _1_(reference group: low) |  |  |  |  |  |  |  |  |  |  |  |
| Intermediate | 0.82 | 0.77 | 0.86 |  | 0.76 | 0.72 | 0.80 |  | 0.81 | 0.77 | 0.87 |
| High | 0.72 | 0.68 | 0.77 |  | 0.68 | 0.64 | 0.71 |  | 0.73 | 0.69 | 0.78 |
| SECON index (reference group: low) |  |  |  |  |  |  |  |  |  |  |  |
| Intermediate | 0.88 | 0.83 | 0.93 |  | 0.84 | 0.80 | 0.88 |  | 0.81 | 0.76 | 0.85 |
| High | 0.66 | 0.62 | 0.71 |  | 0.61 | 0.58 | 0.64 |  | 0.57 | 0.53 | 0.62 |
| **Model 2a:PDCI 2 and UPC index** |  |  |  |  |  |  |  |  |  |  |  |
| PDCI _2_(reference group: low) |  |  |  |  |  |  |  |  |  |  |  |
| Intermediate | 0.83 | 0.77 | 0.88 |  | 0.78 | 0.73 | 0.82 |  | 0.75 | 0.70 | 0.81 |
| High | 0.75 | 0.71 | 0.80 |  | 0.70 | 0.66 | 0.73 |  | 0.69 | 0.65 | 0.74 |
| UPC index (reference group: low) |  |  |  |  |  |  |  |  |  |  |  |
| Intermediate | 0.76 | 0.71 | 0.80 |  | 0.80 | 0.77 | 0.84 |  | 0.71 | 0.67 | 0.75 |
| High | 0.58 | 0.54 | 0.62 |  | 0.58 | 0.55 | 0.62 |  | 0.56 | 0.51 | 0.60 |
| **Model 2b: PDCI 2 and COCI** |  |  |  |  |  |  |  |  |  |  |  |
| PDCI _2_(reference group: low) |  |  |  |  |  |  |  |  |  |  |  |
| Intermediate | 0.82 | 0.77 | 0.88 |  | 0.77 | 0.73 | 0.81 |  | 0.74 | 0.69 | 0.80 |
| High | 0.77 | 0.72 | 0.82 |  | 0.70 | 0.66 | 0.73 |  | 0.69 | 0.64 | 0.73 |
| COCI (reference group: low) |  |  |  |  |  |  |  |  |  |  |  |
| Intermediate | 0.74 | 0.70 | 0.78 |  | 0.76 | 0.73 | 0.80 |  | 0.66 | 0.63 | 0.70 |
| High | 0.52 | 0.49 | 0.55 |  | 0.53 | 0.50 | 0.56 |  | 0.48 | 0.45 | 0.52 |
| **Model 2c: PDCI 2 and SECON index** |  |  |  |  |  |  |  |  |  |  |  |
| PDCI _2_(reference group: low) |  |  |  |  |  |  |  |  |  |  |  |
| Intermediate | 0.82 | 0.77 | 0.87 |  | 0.77 | 0.73 | 0.81 |  | 0.75 | 0.70 | 0.81 |
| High | 0.73 | 0.69 | 0.78 |  | 0.68 | 0.65 | 0.72 |  | 0.69 | 0.65 | 0.74 |
| SECON index (reference group: low) |  |  |  |  |  |  |  |  |  |  |  |
| Intermediate | 0.88 | 0.83 | 0.93 |  | 0.83 | 0.80 | 0.87 |  | 0.80 | 0.76 | 0.85 |
| High | 0.64 | 0.61 | 0.69 |  | 0.58 | 0.55 | 0.61 |  | 0.55 | 0.51 | 0.59 |

IOM, Institute of Medicine; CCI, Charlson comorbidity index; COC, continuity of care; UPC index, usual provider of care index; COCI, continuity of care index; SECON index, sequential Continuity Index; PDCI, provider duration continuity index

Supplementary Table 4 GEE estimations of the effects of the COC measures on the likelihood of hospitalization for any condition by CCI group

|  | **CCI=0** | | |  | **CCI=1** | | |  | **CCI≥2** | | |
| --- | --- | --- | --- | --- | --- | --- | --- | --- | --- | --- | --- |
|  | OR | 95% CI | |  | OR | 95% CI | |  | OR | 95% CI | |
| **Model 1a: PDCI 1 and UPC index** |  |  |  |  |  |  |  |  |  |  |  |
| PDCI _1_(reference group: low) |  |  |  |  |  |  |  |  |  |  |  |
| Intermediate | 0.64 | 0.62 | 0.65 |  | 0.72 | 0.70 | 0.73 |  | 0.62 | 0.61 | 0.64 |
| High | 0.57 | 0.56 | 0.58 |  | 0.69 | 0.67 | 0.70 |  | 0.59 | 0.58 | 0.61 |
| UPC index (reference group: low) |  |  |  |  |  |  |  |  |  |  |  |
| Intermediate | 0.84 | 0.82 | 0.85 |  | 0.75 | 0.74 | 0.77 |  | 0.74 | 0.72 | 0.76 |
| High | 0.58 | 0.56 | 0.59 |  | 0.49 | 0.47 | 0.50 |  | 0.56 | 0.54 | 0.58 |
| **Model 1b:PDCI 1 and COCI** |  |  |  |  |  |  |  |  |  |  |  |
| PDCI _1_(reference group: low) |  |  |  |  |  |  |  |  |  |  |  |
| Intermediate | 0.64 | 0.63 | 0.65 |  | 0.72 | 0.70 | 0.74 |  | 0.63 | 0.61 | 0.65 |
| High | 0.58 | 0.57 | 0.59 |  | 0.70 | 0.68 | 0.71 |  | 0.60 | 0.58 | 0.62 |
| COCI (reference group: low) |  |  |  |  |  |  |  |  |  |  |  |
| Intermediate | 0.84 | 0.82 | 0.86 |  | 0.74 | 0.72 | 0.75 |  | 0.71 | 0.69 | 0.73 |
| High | 0.54 | 0.52 | 0.55 |  | 0.44 | 0.43 | 0.45 |  | 0.48 | 0.47 | 0.50 |
| **Model 1c:PDCI 1 and SECON index** |  |  |  |  |  |  |  |  |  |  |  |
| PDCI _1_(reference group: low) |  |  |  |  |  |  |  |  |  |  |  |
| Intermediate | 0.62 | 0.61 | 0.63 |  | 0.70 | 0.68 | 0.71 |  | 0.62 | 0.60 | 0.64 |
| High | 0.54 | 0.53 | 0.55 |  | 0.65 | 0.64 | 0.66 |  | 0.58 | 0.56 | 0.60 |
| SECON index (reference group: low) |  |  |  |  |  |  |  |  |  |  |  |
| Intermediate | 1.03 | 1.01 | 1.05 |  | 0.89 | 0.87 | 0.91 |  | 0.90 | 0.88 | 0.93 |
| High | 0.74 | 0.72 | 0.75 |  | 0.59 | 0.58 | 0.61 |  | 0.64 | 0.61 | 0.66 |
| **Model 2a:PDCI 2 and UPC index** |  |  |  |  |  |  |  |  |  |  |  |
| PDCI _2_(reference group: low) |  |  |  |  |  |  |  |  |  |  |  |
| Intermediate | 0.73 | 0.71 | 0.74 |  | 0.81 | 0.79 | 0.83 |  | 0.79 | 0.76 | 0.82 |
| High | 0.65 | 0.64 | 0.67 |  | 0.75 | 0.73 | 0.76 |  | 0.70 | 0.67 | 0.72 |
| UPC index (reference group: low) |  |  |  |  |  |  |  |  |  |  |  |
| Intermediate | 0.83 | 0.81 | 0.84 |  | 0.73 | 0.72 | 0.75 |  | 0.72 | 0.70 | 0.74 |
| High | 0.54 | 0.52 | 0.55 |  | 0.46 | 0.45 | 0.47 |  | 0.52 | 0.50 | 0.54 |
| **Model 2b: PDCI 2 and COCI** |  |  |  |  |  |  |  |  |  |  |  |
| PDCI _2_(reference group: low) |  |  |  |  |  |  |  |  |  |  |  |
| Intermediate | 0.73 | 0.71 | 0.74 |  | 0.81 | 0.78 | 0.83 |  | 0.78 | 0.75 | 0.81 |
| High | 0.66 | 0.65 | 0.67 |  | 0.75 | 0.73 | 0.77 |  | 0.69 | 0.67 | 0.72 |
| COCI (reference group: low) |  |  |  |  |  |  |  |  |  |  |  |
| Intermediate | 0.82 | 0.81 | 0.84 |  | 0.72 | 0.71 | 0.74 |  | 0.69 | 0.67 | 0.71 |
| High | 0.50 | 0.49 | 0.51 |  | 0.42 | 0.40 | 0.43 |  | 0.45 | 0.43 | 0.47 |
| **Model 2c: PDCI 2 and SECON index** |  |  |  |  |  |  |  |  |  |  |  |
| PDCI _2_(reference group: low) |  |  |  |  |  |  |  |  |  |  |  |
| Intermediate | 0.72 | 0.71 | 0.74 |  | 0.81 | 0.79 | 0.84 |  | 0.80 | 0.77 | 0.83 |
| High | 0.63 | 0.61 | 0.64 |  | 0.73 | 0.71 | 0.75 |  | 0.70 | 0.68 | 0.72 |
| SECON index (reference group: low) |  |  |  |  |  |  |  |  |  |  |  |
| Intermediate | 1.03 | 1.01 | 1.05 |  | 0.88 | 0.86 | 0.90 |  | 0.90 | 0.88 | 0.93 |
| High | 0.70 | 0.69 | 0.72 |  | 0.57 | 0.55 | 0.58 |  | 0.61 | 0.59 | 0.63 |

IOM, Institute of Medicine; CCI, Charlson comorbidity index; COC, continuity of care; UPC index, usual provider of care index; COCI, continuity of care index; SECON index, sequential Continuity Index; PDCI, provider duration continuity index

Supplementary Table 5 GEE estimations of the effects of the COC measures on the likelihood of hospitalization for avoidable conditions by age group

|  | **Age 20-39 years** | | |  | **Age 40-59 years** | | |  | **Age ≥60 year** | | |
| --- | --- | --- | --- | --- | --- | --- | --- | --- | --- | --- | --- |
|  | OR | 95% CI | |  | OR | 95% CI | |  | OR | 95% CI | |
| **Model 1a: PDCI 1 and UPC index** |  |  |  |  |  |  |  |  |  |  |  |
| PDCI _1_(reference group: low) |  |  |  |  |  |  |  |  |  |  |  |
| Intermediate | 0.82 | 0.71 | 0.96 |  | 0.86 | 0.8 | 0.93 |  | 0.79 | 0.75 | 0.82 |
| High | 0.85 | 0.71 | 1.03 |  | 0.80 | 0.74 | 0.87 |  | 0.70 | 0.67 | 0.73 |
| UPC index (reference group: low) |  |  |  |  |  |  |  |  |  |  |  |
| Intermediate | 0.84 | 0.72 | 0.97 |  | 0.74 | 0.69 | 0.80 |  | 0.82 | 0.79 | 0.85 |
| High | 0.64 | 0.53 | 0.77 |  | 0.61 | 0.55 | 0.66 |  | 0.65 | 0.62 | 0.69 |
| **Model 1b:PDCI 1 and COCI** |  |  |  |  |  |  |  |  |  |  |  |
| PDCI _1_(reference group: low) |  |  |  |  |  |  |  |  |  |  |  |
| Intermediate | 0.83 | 0.71 | 0.97 |  | 0.87 | 0.81 | 0.94 |  | 0.79 | 0.76 | 0.83 |
| High | 0.87 | 0.72 | 1.05 |  | 0.82 | 0.76 | 0.89 |  | 0.71 | 0.68 | 0.74 |
| COCI (reference group: low) |  |  |  |  |  |  |  |  |  |  |  |
| Intermediate | 0.78 | 0.67 | 0.90 |  | 0.70 | 0.65 | 0.76 |  | 0.76 | 0.73 | 0.79 |
| High | 0.59 | 0.49 | 0.72 |  | 0.56 | 0.51 | 0.61 |  | 0.57 | 0.54 | 0.60 |
| **Model 1c:PDCI 1 and SECON index** |  |  |  |  |  |  |  |  |  |  |  |
| PDCI _1_(reference group: low) |  |  |  |  |  |  |  |  |  |  |  |
| Intermediate | 0.81 | 0.70 | 0.94 |  | 0.85 | 0.78 | 0.92 |  | 0.78 | 0.75 | 0.81 |
| High | 0.83 | 0.69 | 1.00 |  | 0.78 | 0.72 | 0.85 |  | 0.69 | 0.66 | 0.72 |
| SECON index (reference group: low) |  |  |  |  |  |  |  |  |  |  |  |
| Intermediate | 0.86 | 0.75 | 1.00 |  | 0.86 | 0.8 | 0.92 |  | 0.84 | 0.81 | 0.87 |
| High | 0.67 | 0.55 | 0.81 |  | 0.59 | 0.55 | 0.65 |  | 0.62 | 0.59 | 0.65 |
| **Model 2a:PDCI 2 and UPC index** |  |  |  |  |  |  |  |  |  |  |  |
| PDCI _2_(reference group: low) |  |  |  |  |  |  |  |  |  |  |  |
| Intermediate | 0.97 | 0.83 | 1.13 |  | 0.87 | 0.8 | 0.95 |  | 0.72 | 0.69 | 0.76 |
| High | 0.8 | 0.65 | 0.97 |  | 0.79 | 0.72 | 0.86 |  | 0.66 | 0.63 | 0.69 |
| UPC index (reference group: low) |  |  |  |  |  |  |  |  |  |  |  |
| Intermediate | 0.84 | 0.72 | 0.97 |  | 0.73 | 0.68 | 0.79 |  | 0.80 | 0.77 | 0.83 |
| High | 0.64 | 0.53 | 0.77 |  | 0.59 | 0.54 | 0.64 |  | 0.61 | 0.58 | 0.64 |
| **Model 2b: PDCI 2 and COCI** |  |  |  |  |  |  |  |  |  |  |  |
| PDCI _2_(reference group: low) |  |  |  |  |  |  |  |  |  |  |  |
| Intermediate | 0.97 | 0.83 | 1.14 |  | 0.87 | 0.80 | 0.95 |  | 0.71 | 0.68 | 0.75 |
| High | 0.81 | 0.67 | 0.99 |  | 0.8 | 0.73 | 0.87 |  | 0.66 | 0.63 | 0.69 |
| COCI (reference group: low) |  |  |  |  |  |  |  |  |  |  |  |
| Intermediate | 0.78 | 0.67 | 0.90 |  | 0.70 | 0.65 | 0.75 |  | 0.74 | 0.72 | 0.77 |
| High | 0.59 | 0.49 | 0.71 |  | 0.54 | 0.50 | 0.59 |  | 0.53 | 0.51 | 0.56 |
| **Model 2c: PDCI 2 and SECON index** |  |  |  |  |  |  |  |  |  |  |  |
| PDCI _2_(reference group: low) |  |  |  |  |  |  |  |  |  |  |  |
| Intermediate | 0.96 | 0.82 | 1.13 |  | 0.87 | 0.8 | 0.94 |  | 0.72 | 0.68 | 0.75 |
| High | 0.78 | 0.64 | 0.95 |  | 0.77 | 0.71 | 0.84 |  | 0.65 | 0.62 | 0.68 |
| SECON index (reference group: low) |  |  |  |  |  |  |  |  |  |  |  |
| Intermediate | 0.86 | 0.75 | 1.00 |  | 0.86 | 0.8 | 0.92 |  | 0.83 | 0.80 | 0.86 |
| High | 0.67 | 0.55 | 0.80 |  | 0.58 | 0.53 | 0.63 |  | 0.59 | 0.56 | 0.62 |

IOM, Institute of Medicine; CCI, Charlson comorbidity index; COC, continuity of care; UPC index, usual provider of care index; COCI, continuity of care index; SECON index, sequential Continuity Index; PDCI, provider duration continuity index

**panel 2014**

***Commonly-used COC indicators measured in 2014***

***Hospitalization measured in 2015***

***Longitudinal COC indicators in 2014 measured from 2007 to 2014***

**panel 2015**

***Commonly-used COC indicators measured in 2015***

***Hospitalization measured in 2016***

***Longitudinal COC indicators in 2015 measured from 2007 to 2015***

**panel 2016**

***Commonly-used COC indicators measured in 2016***

***Longitudinal COC in 2016 measured from 2007 to 2016***

***Hospitalization measured in 2017***

**panel 2017**

***Commonly-used COC indicators measured in 2017***

00

***Longitudinal COC indicators measured in 2017 from 2007 to 2017***

***Hospitalization measured in 2018***

2017

2016

2015

2018

2013

2010

2009

2008

2007

2014

2017

2012

2011

Supplementary Figure 1. Measurement of the time period for care continuity and hospitalization for a typical patient in the subsequent-year model
